# Editorial: Treatment of tick-borne diseases: current status, challenges, and global perspectives

**DOI:** 10.3389/fphar.2024.1366988

**Published:** 2024-03-12

**Authors:** Ketsarin Kamyingkird, Mohamed Z. Sayed-Ahmed, Mohamed Abdo Rizk

**Affiliations:** ^1^ Department of Parasitology, Faculty of Veterinary Medicine, Kasetsart University, Bangkok, Thailand; ^2^ Department of Clinical Pharmacy, College of Pharmacy, Jazan University, Jizan, Saudi Arabia; ^3^ Department of Internal Medicine and Infectious Diseases, Faculty of Veterinary Medicine, Mansoura University, Mansoura, Egypt

**Keywords:** tick-borne diseases, treatment, current status, challenges, global perspective justified

This Research Topic converges different original contributions highlighting animal tick-borne diseases (TBDs), current and emerging treatments, drug discovery, and the analysis of potential drug targets. These contributions shed light on the fields of 1) the discovery of novel, effective, and safe antiparasitic drugs for TBDs affecting animals; 2) investigations into whether the pressures caused by currently used drugs or recently identified drugs against TBDs could induce parasite resistance; and 3) searching for possible drugs against TBDs targets and pathways.

Tick infestations, both in humans and animals, can cause various diseases, including viral, bacterial, protozoal, and parasitic infections. TBDs of veterinary importance, including babesiosis, hepatozoonosis, ehrlichiosis, theileriosis, and anaplasmosis, are still problems in many areas in tropical and subtropical countries. Effective chemotherapeutic drugs are needed for the treatment and control of TBDs. As we already know, the treatment of infections by apicomplexa parasites such as *Babesia* and *Theileria* can be achieved using imidocarb dipropionate and diminazene aceturate at varying doses depending on the actual parasite and host. However, toxicity, low efficacy, and relapse have also been reported. Therefore, novel chemotherapeutic compounds are required. Better control of transmitted vectors such as ticks is also required. Chemotherapeutic drugs for tick control are key to reducing the risk of TBDs in humans and animals. However, chemotherapeutic resistance in ticks has also been reported. It has a huge impact on the efficacy and limitation of compounds for controlling ticks and TBDs. Subsequently, the first contribution to this Research Topic (Obaid et al.) assessed tick resistance to cypermethrin and amitraz using molecular detection of the voltage-gatedsodium channel and octopamine tyramine (OCT/Tyr) genes in the *in vitro R. microplus* larval immersion test in 10% cypermethrin and 12.5% amitraz. They found that there was an SNP at position A-22-C (T-8-P) in the OCT/Tyr gene, which indicated the resistant tick in Pakistan.

For the first time, El-Sayed et al. evaluated the *in vitro*, and *in vivo* efficacy of a synthetic analog of curcumin (FLLL-32) and resveratrol (RVT) on *Babesia* and *Theileria* parasites. Resveratrol inhibits the growth of bovine and equine piroplasms *in vitro*, with *B. bovis* (IC_50_ = 29.51 µM) being the most sensitive treated parasite. Combined treatment consisting of RVT and imidocarb dipropionate is more effective for *B. microti*-infected mice than the currently used antibabesial monotherapies. Thus, RSV may play a protective function in reducing the cardiotoxic effects of azithromycin and closely related medications. The curcumin analog FLLL-32 showed potent inhibitory effects on the *in vitro* growth of *B. bovis*, *B. bigemina*, *B. divergens*, *B. caballi*, and *T. equi* with *B. bovis* (IC_50_ = 9.57 μM) being the most susceptible species. FLLL-32 was shown to inhibit the enzyme S-adenosylhomocysteine hydrolase (SAHH) of *B. bovis* that can increase the SAHH to S-adenosylmethionine (SAM) ratio and block SAM-dependent methyltransferase, which catalyzes the methylation process required for parasite growth. Moreover, the compound showed antibabesial activities against the *in vivo* growth of *B. microti* in infected mice.

Continuing with discovering novel antibabesial drugs, Yu et al. identified two naphthalene-based compounds, DBHCA and DHNA, to be potent anti- *B. microti* candidates. The two naphthalene-based compounds DBHCA and DHNA were identified to target *B. microti* lactate dehydrogenase (*Bm*LDH) and inhibit both the enzyme activity of *Bm*LDH and the growth of *B. microti in vitro*. To better understand the binding properties (association and dissociation) between *Bm*LDH and the naphthalene-based compounds, Yu et al. demonstrated surface plasmon resonance analysis. The results demonstrated that DHNA has a lower *K*_D_ value to *Bm*LDH (3.766 × 10^−5^ M) in contrast to a higher value for DBHCA (3.988 × 10^−8^ M). A comparison of the kinetic parameters [association constant (*k*_a_) and dissociation constant (*k*_d_) values] reveals that DBHCA can bind the target faster than DHNA, while the complex of DHNA with the target dissociates slower than that of DBHCA.

Additionally, cytotoxicity tests of DBHCA and DHNA in the Vero cell line further demonstrated that DHNA has a higher selectivity index than DBHCA between *B. microti* and Vero cells. These findings provide some theoretical basis for renewed structure-based development of the two naphthalene-based compounds as novel anti-*Babesia* agents for the treatment of human babesiosis.

As editors of this Research Topic, we would like to acknowledge all authors who have contributed high-quality original articles. We hope that the reader will find in this Research Topic a useful reference for the state of the art of knowledge of treatment of tick-borne diseases across two broad aspects: 1) the discovery of novel, effective, and safe anti-TBD drugs and 2) the search for possible drugs against TBDs targets and pathways.

